# Supplementary figures and images for: Physiological TLR4 regulation in human fetal membranes as an explicative mechanism of a pathological preterm case
Source: eLife. 2022 Feb 4;11:e71521. doi: 10.7554/eLife.71521 (PMC8816379; doi:10.7554/eLife.71521)

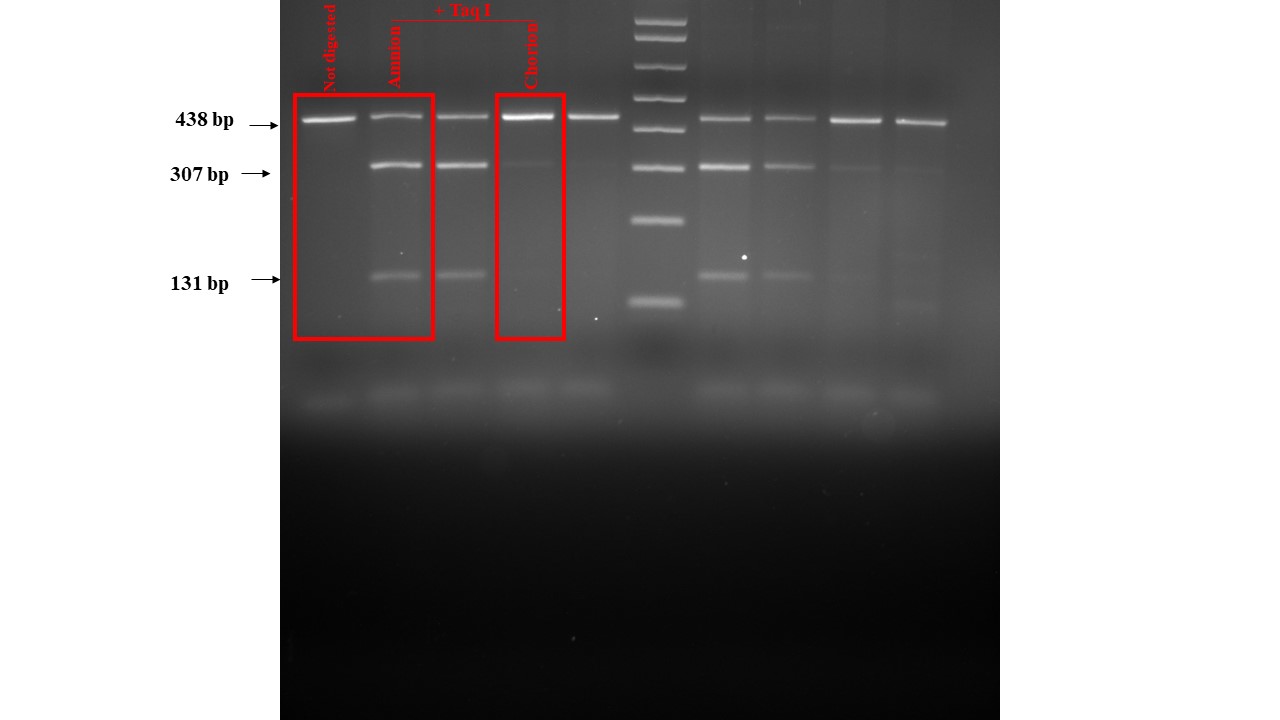

Supplement: Figure 4—source data 2. [file elife-71521-fig4-data2.zip › Figure4_source data 2.jpg]
