# Supplementary material for: Physiological TLR4 regulation in human fetal membranes as an explicative mechanism of a pathological preterm case
Source: eLife. 2022 Feb 4;11:e71521. doi: 10.7554/eLife.71521 (PMC8816379; doi:10.7554/eLife.71521)
Supplement: Supplementary file 1. [file elife-71521-supp1.docx]

**Supplementary file 1a :**

**Perinatal characteristics of the enrolled patients.**

| **Perinatal characteristics** | **n=9** |
| --- | --- |
| **Maternal Age (years)** | 32.33 ± 1.76 |
| **Gestational age at delivery (**Amenorrhea weeks) | 39.10 ± 0.08 |
| **Body Mass Index, BMI** | 23.75 ± 0.95 |
| **Child sexe** | 5F, 4M |
| **Birth weight (g)** | 3351 ± 129 |

**Supplementary file 1b:**

**Primer sequences used for the PCR.**

| **Human Gene** | **Sequence (5’🡪3’)** | **Product length (bp)** | **NCBI**  **Reference** |
| --- | --- | --- | --- |
| hTLR4-S | ACCAAGAACCTGGACCTGAG | 181 | NM_138554 |
| hTLR4-A | TCTGGATGGGGTTTCCTGTC |  |  |
| hRPLP0-S | AGGCTTTAGGTATCACCACT | 219 | NM_053275 |
| hRPLP0-A | GCAGAGTTTCCTCTGTGATA |  |  |
| hRPS17-S | TGCGAGGAGATCGCCATTATC | 170 | NM_001021 |
| hRPS17-A | AAGGCTGAGACCTCAGGAAC |  |  |
| hpri-125B1-S | CGAACAGAAATTGCCTGTCATTC | 175 | [NR_029671](http://www.ncbi.nlm.nih.gov/nuccore/NR_029671) |
| hpri-125B1-A | TTCCACCAAATTTCCAGGATGC |  |  |
| hpri-LET7A2-S | AGACTAACTTGTAATTTCCCTGC | 190 | [NR_029477](http://www.ncbi.nlm.nih.gov/nuccore/NR_029477) |
| hpri-LET7A2-A | AGGCCTGGAGGAATCATGATC |  |  |
| hpri-LET7A1-S | TTCCTGTGGTGCTCAACTGTG | 200 | NR_029476 |
| hpri-LET7A1-A | TGTACAATTAGTTAACTGACTTTC |  |  |

**Supplementary file 1c:**

**List of specific genes extracted from ZAM zone after a transcriptomic analysis**

| **log2 FC<2.8**   \| A2M \| \| --- \| \| AADAC \| \| ABCA1 \| \| ABP1 \| \| ACVRL1 \| \| ADA \| \| ADAM12 \| \| ADAM19 \| \| ADAMTS1 \| \| ADAMTS4 \| \| ADAMTS5 \| \| ADAMTS8 \| \| ADCY1 \| \| AGT \| \| AKAP12 \| \| ALDH2 \| \| AMIGO2 \| \| AMZ1 \| \| ANKRD1 \| \| ANTXR1 \| \| APOA1 \| \| APOBEC2 \| \| APOC1 \| \| APOE \| \| AQPEP \| \| AR \| \| ARG1 \| \| ASPHD2 \| \| ASS1 \| \| ATP13A4 \| \| AXDND1 \| \| BCAR4 \| \| BCORL1 \| \| BIN2 \| \| BMP8A \| \| C11orf20 \| \| C11orf86 \| \| C12orf42 \| \| C15orf48 \| \| C1orf115 \| \| C1orf130 \| \| C1QTNF1 \| \| C2CD4B \| \| C2orf72 \| \| C4BPB \| \| C4orf26 \| \| C5orf46 \| \| C7orf58 \| \| C8orf31 \| \| CABLES1 \| \| CACNA1A \| \| CACNA1C \| \| CCNO \| \| CD248 \| \| CD302 \| \| CD52 \| \| CDA \| \| CDCA7 \| \| CDH16 \| \| CDO1 \| \| CEACAM1 \| \| CEACAM3 \| \| CEBPA \| \| CFHR3 \| \| CHST2 \| \| CILP2 \| \| CKMT2 \| \| CLDN19 \| \| CNR1 \| \| COL14A1 \| \| COL27A1 \| \| CORO2A \| \| COTL1 \| \| CPE \| \| CPZ \| \| CSF2RB \| \| CSH1 \| \| CSH2 \| \| CSHL1 \| \| CSRP2 \| \| CTAG1A \| \| CTNND2 \| \| CTSH \| \| CTSL1 \| \| CTSL1P8 \| \| CXCL12 \| \| CXCL14 \| \| CXCR6 \| \| CYP11A1 \| \| CYP26A1 \| \| CYP4B1 \| \| DAB2IP \| \| DENND2A \| \| DFNA5 \| \| DHRS3 \| \| DIO2 \| \| DIRC3 \| \| DISC1 \| \| DLK2 \| \| DLL1 \| \| DLX4 \| \| DLX5 \| \| DLX6 \| \| DNAJC6 \| \| DOCK4 \| \| DPP6 \| \| DSCR8 \| \| DZIP1 \| \| E2F2 \| \| EBF1 \| \| EBI3 \| \| ECM2 \| \| EDNRB \| \| EEPD1 \| \| EFHD1 \| \| EFNB1 \| \| ELTD1 \| \| ENG \| \| ENTPD2 \| \| ENTPD3 \| \| ENTPD8 \| \| EPHB2 \| \| ERVMER34-1 \| \| ESAM \| \| ESR1 \| \| ESYT3 \| \| EXOC3L4 \| \| FA2H \| \| FABP7 \| \| FADS1 \| \| FADS3 \| \| FAM105A \| \| FAM153A \| \| FAM153B \| \| FAM43A \| \| FAM78A \| \| FAM83D \| \| FBLN1 \| \| FBXL16 \| \| FBXO32 \| \| FGF2 \| \| FGFBP1 \| \| FGFR2 \| \| FHOD3 \| \| FIGNL2 \| \| FILIP1L \| \| FLJ13744 \| \| FLJ16779 \| \| FLJ30901 \| \| FLJ31356 \| \| FLJ43663 \| \| FLT1 \| \| FOLR1 \| \| FOXO1 \| \| GCM1 \| \| GDA \| \| GDF15 \| \| GDNF \| \| GDPD3 \| \| GEM \| \| GH1 \| \| GJC1 \| \| GJD3 \| \| GKN1 \| \| GLDC \| \| GLIPR1 \| \| GNLY \| \| GPC4 \| \| GPR116 \| \| GPR32 \| \| GPR78 \| \| GPX3 \| \| GRIK2 \| \| GTSF1 \| \| GYLTL1B \| \| HAS3 \| \| HERC6 \| \| HES2 \| \| HEXB \| \| HEY1 \| \| HJURP \| \| HMGB3 \| \| HMGB3P1 \| \| HMHA1 \| \| HN1 \| \| HOXA11 \| \| HOXA7 \| \| HPCAL1 \| \| HPGD \| \| HS3ST2 \| \| HS6ST1 \| \| HSD3B1 \| \| HSPA12B \| \| HTRA4 \| \| HYAL4 \| \| ICAM2 \| \| IGDCC3 \| \| IGSF23 \| \| IL13RA2 \| \| IL15 \| \| IL17D \| \| IL18BP \| \| IL18R1 \| \| IL1RAP \| \| IL1RL1 \| \| IL20RB \| \| IL2RB \| \| IL33 \| \| IL6ST \| \| INHA \| \| INHBA \| \| ISM2 \| \| ITGA1 \| \| ITGAD \| \| ITGB3 \| \| ITLN1 \| \| ITLN2 \| \| JAM2 \| \| JAZF1 \| \| KAL1 \| \| KCNA4 \| \| KCNJ16 \| \| KCNJ2 \| \| KCNK12 \| \| KIAA1324 \| \| KIF21A \| \| KISS1R \| \| KITLG \| \| KLHL5 \| \| KLK3 \| \| KLRC1 \| \| KLRC4 \| \| KLRG2 \| \| KRT37 \| \| LAIR2 \| \| LAMB1 \| \| LAMB4 \| \| LDLR \| \| LGALS14 \| \| LGSN \| \| LIFR \| \| LIMS3L \| \| LIN28B \| \| LINC00162 \| \| LINC00221 \| \| LINC00473 \| \| LINGO2 \| \| LIPG \| \| LITAF \| \| LOC100128098 \| \| LOC100130111 \| \| LOC100132735 \| \| LOC100302650 \| \| LOC100505495 \| \| LOC100506328 \| \| LOC100506580 \| \| LOC100506783 \| \| LOC100507033 \| \| LOC100507140 \| \| LOC100507351 \| \| LOC100507410 \| \| LOC100507566 \| \| LOC100507639 \| \| LOC100509091 \| \| LOC100509498 \| \| LOC100652823 \| \| LOC144817 \| \| LOC145820 \| \| LOC219731 \| \| LOC283404 \| \| LOC284561 \| \| LOC339524 \| \| LOC340340 \| \| LOC388242 \| \| LOC388630 \| \| LOC389634 \| \| LOC644366 \| \| LOC644686 \| \| LOC645722 \| \| LOC650226 \| \| LOC729178 \| \| LOC729680 \| \| LUM \| \| MAGEA8 \| \| MAPT \| \| MARC1 \| \| MCAM \| \| MFAP3L \| \| MFSD2A \| \| MGAT5B \| \| MGP \| \| MGST1 \| \| MIAT \| \| MIR205HG \| \| MLC1 \| \| MMP11 \| \| MMP7 \| \| MPDZ \| \| MYCL1 \| \| MYO16 \| \| NAV3 \| \| NBLA00301 \| \| NDC80 \| \| NFASC \| \| NFE2L3 \| \| NIM1 \| \| NKD2 \| \| NKG7 \| \| NLGN4X \| \| NLGN4Y \| \| NLRP7 \| \| NOTUM \| \| NRCAM \| \| NRIP1 \| \| NTN1 \| \| NTN4 \| \| OGDHL \| \| OLR1 \| \| OOEP \| \| OSR2 \| \| OXGR1 \| \| PAGE4 \| \| PALM3 \| \| PAPLN \| \| PAPPA \| \| PAPPA2 \| \| PAPSS2 \| \| PARD6G \| \| PARM1 \| \| PCDHB10 \| \| PCDHB9 \| \| PCOLCE2 \| \| PDCD1LG2 \| \| PDE6H \| \| PDE8B \| \| PDGFRL \| \| PDZD2 \| \| PDZK1IP1 \| \| PGF \| \| PKN1 \| \| PLA2G7 \| \| PLA2R1 \| \| PLAC8 \| \| PLCE1 \| \| PLEKHH2 \| \| PLIN1 \| \| PLXDC1 \| \| PNMAL1 \| \| PNPLA3 \| \| POLR1C \| \| PPAP2A \| \| PPAPDC3 \| \| PPARG \| \| PPP4R4 \| \| PRB3 \| \| PRDM1 \| \| PRG2 \| \| PRKCE \| \| PROK1 \| \| PROS1 \| \| PSG1 \| \| PSG10P \| \| PSG2 \| \| PSG3 \| \| PSG5 \| \| PSG6 \| \| PSG8 \| \| PTGDS \| \| PTGER3 \| \| PTN \| \| PTPN13 \| \| PVR \| \| Q6TXI9 \| \| RAB15 \| \| RAB3B \| \| RASGRF2 \| \| RASL11B \| \| RBM47 \| \| RBPMS \| \| REN \| \| REPS2 \| \| RGN \| \| RGPD1 \| \| RGS16 \| \| RNF150 \| \| RRAD \| \| S100P \| \| SCARB1 \| \| SCGB1D1 \| \| SCUBE1 \| \| SCUBE2 \| \| SEMA5B \| \| SERPINB11 \| \| SERPINB9 \| \| SFRP1 \| \| SH3BP4 \| \| SH3BP5 \| \| SLC16A6 \| \| SLC22A18AS \| \| SLC25A15 \| \| SLC44A5 \| \| SLC4A7 \| \| SLC6A2 \| \| SLC6A4 \| \| SLCO2A1 \| \| SNAI1 \| \| SNORD115-1 \| \| SNORD115-23 \| \| SNORD115-32 \| \| SNORD115-4 \| \| SNX10 \| \| SOAT1 \| \| SOD3 \| \| SOHLH2 \| \| SORBS1 \| \| SPATA22 \| \| SPDYE5 \| \| SPINK5 \| \| SPOCD1 \| \| SPRR2G \| \| SRPX \| \| SSH2 \| \| ST3GAL5 \| \| ST8SIA1 \| \| STAB2 \| \| STON1-GTF2A1L \| \| STRA6 \| \| SULF1 \| \| SULF2 \| \| SUN3 \| \| SV2B \| \| SYN3 \| \| SYNPO \| \| SYNPO2L \| \| SYT12 \| \| TAC3 \| \| TBC1D4 \| \| TCF7L1 \| \| TCF7L2 \| \| TIMP4 \| \| TKTL1 \| \| TLE2 \| \| TLE6 \| \| TLR4 \| \| TM4SF5 \| \| TMEM108 \| \| TMEM132B \| \| TMEM204 \| \| TMEM59L \| \| TNFAIP2 \| \| TNFSF10 \| \| TPRXL \| \| TPST1 \| \| TRH \| \| TRIM14 \| \| TRIM64 \| \| TSKU \| \| TSNARE1 \| \| TSPAN5 \| \| TSPAN8 \| \| TTR \| \| UBD \| \| UCHL1 \| \| VAV3 \| \| VIPR2 \| \| VIT \| \| VWCE \| \| WDR86 \| \| WNK2 \| \| WNT10B \| \| WNT6 \| \| XAGE3 \| \| XAGE5 \| \| XLOC_001595 \| \| XLOC_003286 \| \| XLOC_003595 \| \| XLOC_006705 \| \| XLOC_006781 \| \| XLOC_007052 \| \| XLOC_007358 \| \| XLOC_007370 \| \| XLOC_007689 \| \| XLOC_007875 \| \| XLOC_008696 \| \| XLOC_010962 \| \| XLOC_011609 \| \| XLOC_011645 \| \| XLOC_012981 \| \| XLOC_013541 \| \| XLOC_014351 \| \| XLOC_l2_000384 \| \| XLOC_l2_004317 \| \| XLOC_l2_007986 \| \| XLOC_l2_009139 \| \| XLOC_l2_009292 \| \| XLOC_l2_009441 \| \| XLOC_l2_009811 \| \| XLOC_l2_010056 \| \| XLOC_l2_012745 \| \| ZFP57 \| \| ZNF114 \| | **log2 FC>2.8**   \| ABCC2 \| \| --- \| \| ABHD11-AS1 \| \| ADRB2 \| \| AFF2 \| \| AHNAK2 \| \| ANK3 \| \| ANO1-AS2 \| \| AP1S3 \| \| ARHGEF37 \| \| ATP12A \| \| AVPR1A \| \| BCKDHB \| \| BCL11A \| \| BSPRY \| \| BZRAP1 \| \| C12orf28 \| \| C14orf34 \| \| C15orf41 \| \| C6orf15 \| \| C7orf41 \| \| C9orf167 \| \| CAMK1D \| \| CAMSAP3 \| \| CCDC165 \| \| CCNB3 \| \| CDHR4 \| \| CDK5R1 \| \| CLDN3 \| \| CLEC1B \| \| CLU \| \| CPS1 \| \| CSF3 \| \| CTSC \| \| CUBN \| \| CYP21A2 \| \| CYP27C1 \| \| DEFB103B \| \| DGKB \| \| DNAJA4 \| \| DNAJC27-AS1 \| \| DNER \| \| DSG1 \| \| DST \| \| DUOXA1 \| \| EMP1 \| \| ENPP5 \| \| EPB41 \| \| EPHA4 \| \| FBXO2 \| \| FBXO41 \| \| FIGN \| \| FLJ25917 \| \| FZD1 \| \| GABBR1 \| \| GABRB1 \| \| GABRP \| \| GAS6 \| \| GJC2 \| \| GSTO2 \| \| GUCY1A3 \| \| HIVEP1 \| \| HOXA2 \| \| HPN \| \| ID4 \| \| ITPR3 \| \| IZUMO2 \| \| KCNJ15 \| \| KCNMA1 \| \| KCTD4 \| \| KIAA1244 \| \| KLHDC7B \| \| KLK5 \| \| KLK6 \| \| KRT27 \| \| KRT6B \| \| LEP \| \| LGALS7 \| \| LGR6 \| \| LINC00239 \| \| LOC100131662 \| \| LOC100287314 \| \| LOC100505904 \| \| LOC283070 \| \| LOC340335 \| \| LOC645638 \| \| LOC648149 \| \| MBOAT1 \| \| MTL5 \| \| NCRNA00185 \| \| NDRG2 \| \| NOD2 \| \| NR3C2 \| \| NTRK3 \| \| OCIAD2 \| \| OR2T10 \| \| OR2T2 \| \| PDE4D \| \| PGBD3 \| \| PLCD1 \| \| POU3F3 \| \| PP12719 \| \| PPP1R13B \| \| PPP2R2B \| \| PRPH2 \| \| PTHLH \| \| RASGRF1 \| \| RGS9BP \| \| RNF222 \| \| RSPH1 \| \| S100A4 \| \| SAA2 \| \| SCRN1 \| \| SEMA4D \| \| SERPINB5 \| \| SERPINB7 \| \| SFN \| \| SGPP2 \| \| SLIT2 \| \| SORD \| \| SORT1 \| \| SOX9 \| \| SPACA4 \| \| SPRR2A \| \| TAS1R3 \| \| TMEM159 \| \| TMEM52 \| \| TMSB15B \| \| TOB1 \| \| TRIM29 \| \| TUBB2B \| \| UPK1B \| \| UPK3B \| \| WFDC2 \| \| WNT2B \| \| XLOC_001317 \| \| XLOC_007116 \| \| XLOC_010945 \| \| XLOC_012199 \| \| XLOC_012665 \| \| XLOC_014194 \| \| XLOC_l2_001947 \| \| XLOC_l2_006079 \| \| XLOC_l2_007543 \| \| XLOC_l2_010751 \| \| ZNF474 \| |
| --- | --- | --- | --- | --- | --- | --- | --- | --- | --- | --- | --- | --- | --- | --- | --- | --- | --- | --- | --- | --- | --- | --- | --- | --- | --- | --- | --- | --- | --- | --- | --- | --- | --- | --- | --- | --- | --- | --- | --- | --- | --- | --- | --- | --- | --- | --- | --- | --- | --- | --- | --- | --- | --- | --- | --- | --- | --- | --- | --- | --- | --- | --- | --- | --- | --- | --- | --- | --- | --- | --- | --- | --- | --- | --- | --- | --- | --- | --- | --- | --- | --- | --- | --- | --- | --- | --- | --- | --- | --- | --- | --- | --- | --- | --- | --- | --- | --- | --- | --- | --- | --- | --- | --- | --- | --- | --- | --- | --- | --- | --- | --- | --- | --- | --- | --- | --- | --- | --- | --- | --- | --- | --- | --- | --- | --- | --- | --- | --- | --- | --- | --- | --- | --- | --- | --- | --- | --- | --- | --- | --- | --- | --- | --- | --- | --- | --- | --- | --- | --- | --- | --- | --- | --- | --- | --- | --- | --- | --- | --- | --- | --- | --- | --- | --- | --- | --- | --- | --- | --- | --- | --- | --- | --- | --- | --- | --- | --- | --- | --- | --- | --- | --- | --- | --- | --- | --- | --- | --- | --- | --- | --- | --- | --- | --- | --- | --- | --- | --- | --- | --- | --- | --- | --- | --- | --- | --- | --- | --- | --- | --- | --- | --- | --- | --- | --- | --- | --- | --- | --- | --- | --- | --- | --- | --- | --- | --- | --- | --- | --- | --- | --- | --- | --- | --- | --- | --- | --- | --- | --- | --- | --- | --- | --- | --- | --- | --- | --- | --- | --- | --- | --- | --- | --- | --- | --- | --- | --- | --- | --- | --- | --- | --- | --- | --- | --- | --- | --- | --- | --- | --- | --- | --- | --- | --- | --- | --- | --- | --- | --- | --- | --- | --- | --- | --- | --- | --- | --- | --- | --- | --- | --- | --- | --- | --- | --- | --- | --- | --- | --- | --- | --- | --- | --- | --- | --- | --- | --- | --- | --- | --- | --- | --- | --- | --- | --- | --- | --- | --- | --- | --- | --- | --- | --- | --- | --- | --- | --- | --- | --- | --- | --- | --- | --- | --- | --- | --- | --- | --- | --- | --- | --- | --- | --- | --- | --- | --- | --- | --- | --- | --- | --- | --- | --- | --- | --- | --- | --- | --- | --- | --- | --- | --- | --- | --- | --- | --- | --- | --- | --- | --- | --- | --- | --- | --- | --- | --- | --- | --- | --- | --- | --- | --- | --- | --- | --- | --- | --- | --- | --- | --- | --- | --- | --- | --- | --- | --- | --- | --- | --- | --- | --- | --- | --- | --- | --- | --- | --- | --- | --- | --- | --- | --- | --- | --- | --- | --- | --- | --- | --- | --- | --- | --- | --- | --- | --- | --- | --- | --- | --- | --- | --- | --- | --- | --- | --- | --- | --- | --- | --- | --- | --- | --- | --- | --- | --- | --- | --- | --- | --- | --- | --- | --- | --- | --- | --- | --- | --- | --- | --- | --- | --- | --- | --- | --- | --- | --- | --- | --- | --- | --- | --- | --- | --- | --- | --- | --- | --- | --- | --- | --- | --- | --- | --- | --- | --- | --- | --- | --- | --- | --- | --- | --- | --- | --- | --- | --- | --- | --- | --- | --- | --- | --- | --- | --- | --- | --- | --- | --- | --- | --- | --- | --- | --- | --- | --- | --- | --- | --- | --- | --- | --- | --- | --- | --- | --- | --- | --- | --- | --- | --- | --- | --- | --- | --- | --- | --- | --- | --- | --- | --- | --- | --- | --- | --- | --- | --- | --- | --- | --- | --- | --- | --- | --- | --- | --- | --- | --- | --- | --- | --- | --- | --- | --- | --- | --- | --- | --- | --- | --- | --- | --- | --- | --- | --- | --- | --- | --- | --- | --- | --- | --- | --- | --- | --- | --- | --- | --- | --- | --- | --- | --- | --- | --- | --- | --- | --- | --- | --- | --- | --- | --- | --- | --- | --- | --- | --- | --- | --- | --- | --- | --- | --- | --- | --- | --- | --- | --- | --- | --- | --- | --- | --- | --- | --- | --- | --- | --- | --- | --- | --- | --- | --- | --- | --- | --- | --- | --- | --- | --- | --- | --- | --- | --- | --- | --- | --- | --- |

**Supplementary file 1d:**

**List of jointly hypermethylated genes in ZAM Choriodecidua and over expressed in ZAM Amnion**

| AP1S3 |
| --- |
| BZRAP1 |
| CDK5R1 |
| DNAJA4 |
| EPB41 |
| FBXO41 |
| FIGN |
| FZD1 |
| GABRB1 |
| GABRP |
| GJC2 |
| LEP |
| NDRG2 |
| NR3C2 |
| POU3F3 |
| PPP1R13B |
| RNF222 |
| RSPH1 |
| SEMA4D |
| SERPINB7 |
| SORD |
| SOX9 |
| TOB1 |
| TRIM29 |
| WFDC2 |
| WNT2B |

**Supplementary file 1e:**

**List of disease mesh-term associated with hypermethylated genes in ZAM Choriodecidua and over expressed in ZAM Amnion**

| **MeSH-Term** | **MeSH-Term id(s)** | **p-value** |
| --- | --- | --- |
| Disease Attributes | C23.550.291 | 8,17E-05 |
| Virilism | C23.888.971 | 1,52E-04 |
| Genetic Predisposition to Disease | C23.550.291.687.500 | 3,24E-04 |
| Disease Susceptibility | C23.550.291.687 | 4,59E-04 |
| Disease Progression | C23.550.291.656 | 4,84E-04 |
| Osteoporotic Fractures | C26.404.545 | 8,30E-04 |
| Endocrine Gland Neoplasms | C19.344, C04.588.322 | 8,85E-04 |
| Gliosis | C23.550.369 | 1,26E-03 |
| Testicular Diseases | C12.294.829 | 1,49E-03 |
| alpha-Thalassemia | C16.320.070.875.100, C15.378.071.141.150.875.100 | 1,50E-03 |
| Pancreatic Neoplasms | C19.344.421, C06.301.761, C06.689.667, C04.588.274.761, C04.588.322.475 | 1,50E-03 |
| Brain Neoplasms | C10.228.140.211, C10.551.240.250, C04.588.614.250.195 | 1,50E-03 |
| Anuria | C13.351.968.934.070, C13.351.968.419.078, C12.777.419.078, C12.777.934.141 | 1,65E-03 |
| Foot Deformities | C05.330 | 1,76E-03 |
| Lymphatic Metastasis | C23.550.727.650.560, C04.697.650.560 | 1,78E-03 |
| Pancreatic Diseases | C06.689 | 1,91E-03 |
| Skin Neoplasms | C17.800.882, C04.588.805 | 1,93E-03 |
| Neoplasms, Germ Cell and Embryonal | C04.557.465 | 2,06E-03 |
| Hyperandrogenism | C16.131.939.316.129.750, C12.706.316.064.500, C13.351.875.253.064.500, C19.391.119.129.750, C13.351.875.253.129.750, C16.131.939.316.064.500, C19.391.119.064.500, C12.706.316.129.750 | 2,14E-03 |
| Dementia | C10.228.140.380 | 2,16E-03 |
| Kwashiorkor | C18.654.521.500.708.626.505 | 2,30E-03 |
| Neurofibroma | C04.557.580.600.580 | 2,37E-03 |
| Cryptorchidism | C12.294.829.258, C16.131.939.258, C19.391.829.258, C12.706.258 | 2,46E-03 |
| Genital Neoplasms, Male | C12.758.409, C04.588.945.440, C12.294.260 | 2,55E-03 |
| Nerve Sheath Neoplasms | C04.557.580.600 | 2,62E-03 |
| Central Nervous System Neoplasms | C04.588.614.250, C10.551.240 | 2,68E-03 |
| Cardio-Renal Syndrome | C13.351.968.419.780.400, C14.280.434.156, C12.777.419.780.400 | 2,75E-03 |
| Sarcoma | C04.557.450.795 | 3,32E-03 |
| Urogenital Abnormalities | C16.131.939 | 3,38E-03 |
| Nervous System Neoplasms | C10.551 | 3,39E-03 |
| Nervous System Neoplasms | C04.588.614 | 3,42E-03 |
| Autoimmune Diseases | C20.111 | 3,44E-03 |
| Urogenital Abnormalities | C12.706 | 3,45E-03 |
| Mandibular Fractures | C26.404.750.467.441, C26.260.275.500.400.255 | 3,46E-03 |
| Mandibular Diseases | C05.500.607 | 3,62E-03 |
| Uremia | C13.351.968.419.936, C12.777.419.936 | 3,92E-03 |
| Jaw Fractures | C26.404.750.467, C26.260.275.500.400 | 4,01E-03 |
| Brain Infarction | C14.907.253.855.200, C10.228.140.300.775.200, C10.228.140.300.150.477, C14.907.253.092.477 | 4,03E-03 |
| Female Athlete Triad Syndrome | C05.116.198.579.304, C19.391.240 | 4,11E-03 |
| Mandibular Diseases | C07.320.610 | 4,14E-03 |
| Neoplasm Metastasis | C23.550.727.650, C04.697.650 | 4,35E-03 |
| Prostatic Neoplasms | C12.758.409.750, C12.294.260.750, C04.588.945.440.770, C12.294.565.625 | 4,49E-03 |
| Brain Diseases | C10.228.140 | 4,55E-03 |
| Sertoli Cell-Only Syndrome | C12.294.365.700.754 | 4,60E-03 |
| Pathologic Processes | C23.550 | 4,63E-03 |
| Osteophyte | C05.116.540.310.800 | 4,72E-03 |
| Temporomandibular Joint Disorders | C05.500.607.221.897, C07.320.610.291.897, C05.550.905, C05.651.243.897, C07.678 | 4,75E-03 |
| Pleural Effusion | C08.528.652 | 4,76E-03 |
| Adrenocortical Adenoma | C19.344.078.265.500, C19.053.347.500.500, C19.053.098.265.500, C04.588.322.078.265.500 | 4,80E-03 |
| Pseudohypoaldosteronism | C16.320.565.861.770, C13.351.968.419.815.770, C12.777.419.815.770, C18.452.648.861.770 | 4,84E-03 |
| Premenstrual Syndrome | C23.550.568.968 | 4,84E-03 |
| 46, XX Disorders of Sex Development | C12.706.316.064, C19.391.119.064, C13.351.875.253.064, C16.131.939.316.064 | 4,85E-03 |
| Craniomandibular Disorders | C05.500.607.221, C07.320.610.291, C05.651.243 | 4,96E-03 |
| Osteoporosis | C05.116.198.579 | 5,04E-03 |
| Prostatic Diseases | C12.294.565 | 5,29E-03 |
| alpha-Thalassemia | C16.320.365.826.100, C15.378.420.826.100 | 5,29E-03 |
| Neurodegenerative Diseases | C10.574 | 5,32E-03 |
| Tracheomalacia | C17.300.182.895.500, C16.131.621.953.500, C08.907.796.500, C05.182.895.500 | 5,48E-03 |
| Tracheobronchomalacia | C08.907.796 | 5,48E-03 |
| Anhedonia | C23.888.592.604.039, C10.597.606.057 | 5,61E-03 |
| Heart Septal Defects, Ventricular | C16.131.240.400.560.540, C14.280.400.560.540, C14.240.400.560.540 | 5,87E-03 |
| Neurilemmoma | C04.557.580.625.650.595 | 6,08E-03 |
| Genital Diseases, Male | C12.294 | 6,11E-03 |
| Infertility, Male | C12.294.365.700 | 6,15E-03 |
| Pseudotumor Cerebri | C10.228.140.631.750 | 6,16E-03 |
| Endocrine System Diseases | C19 | 6,43E-03 |
| Brain Ischemia | C14.907.253.092, C10.228.140.300.150 | 6,44E-03 |
| Tooth Diseases | C07.793 | 6,50E-03 |
| Respiratory Tract Diseases | C08 | 6,65E-03 |
| Cholestasis | C06.130.120.135 | 6,80E-03 |
| Kidney Failure, Chronic | C13.351.968.419.780.750.500, C12.777.419.780.750.500 | 6,82E-03 |
| Tracheobronchomalacia | C16.131.621.953, C17.300.182.895, C05.182.895 | 6,84E-03 |
| Urinary Tract Infections | C01.539.895, C13.351.968.892, C12.777.892 | 6,85E-03 |
| Maxillofacial Injuries | C26.260.275.500 | 7,02E-03 |
| Autoimmune Diseases of the Nervous System | C20.111.258, C10.114 | 7,16E-03 |
| Encephalomyelitis, Autoimmune, Experimental | C20.111.258.625.300, C10.114.703.300, C10.314.350.250, C10.228.140.695.562.250 | 7,19E-03 |
| Immune System Diseases | C20 | 7,22E-03 |
| Ossification of Posterior Longitudinal Ligament | C05.116.900.480, C23.550.751.500 | 7,31E-03 |
| Cerebrovascular Disorders | C10.228.140.300, C14.907.253 | 7,69E-03 |
| Osteochondritis | C17.300.182.520, C05.182.520 | 7,77E-03 |
| Chondrosarcoma | C04.557.450.795.300, C04.557.450.565.280 | 7,78E-03 |
| Pelvic Inflammatory Disease | C01.539.635.500 | 8,08E-03 |
| Neurilemmoma | C04.557.580.600.610.595, C04.557.465.625.650.595 | 8,20E-03 |
| Refeeding Syndrome | C18.654.521.687 | 8,21E-03 |
| Acanthosis Nigricans | C17.800.621.430.530.100 | 8,24E-03 |
| Urogenital Neoplasms | C12.758 | 8,25E-03 |
| Carcinoma, Basal Cell | C04.557.470.565.165, C04.557.470.200.165 | 8,36E-03 |
| Nephritis, Interstitial | C12.777.419.570.643, C13.351.968.419.570.643 | 8,50E-03 |
| Nervous System Autoimmune Disease, Experimental | C10.114.703, C20.111.258.625 | 8,55E-03 |
| Teratocarcinoma | C04.557.465.900 | 8,59E-03 |
| Hyperphosphatemia | C18.452.750.199 | 8,72E-03 |
| Pelvic Infection | C01.539.635 | 8,72E-03 |
| Pleural Diseases | C08.528 | 8,98E-03 |
| Demyelinating Autoimmune Diseases, CNS | C10.228.140.695.562 | 9,11E-03 |
| Neuroendocrine Tumors | C04.557.580.625.650 | 9,17E-03 |
| Central Nervous System Diseases | C10.228 | 9,19E-03 |
| Hereditary Autoinflammatory Diseases | C17.800.827.368 | 9,39E-03 |
| Neuroendocrine Tumors | C04.557.465.625.650 | 9,42E-03 |
| Heart Failure, Systolic | C14.280.434.676 | 9,56E-03 |
| TDP-43 Proteinopathies | C10.574.950, C18.452.845.800 | 9,75E-03 |
| Demyelinating Autoimmune Diseases, CNS | C10.314.350 | 9,83E-03 |
| Carcinogenesis | C04.697.098, C23.550.727.098 | 9,88E-03 |
| Chromosome Inversion | C23.550.210.190 | 9,88E-03 |
| Esophageal and Gastric Varices | C06.552.494.414, C06.405.117.240 | 9,90E-03 |
| Protozoan Infections, Animal | C03.752.625, C03.701.688, C22.674.710 | 9,94E-03 |

**Supplementary file 1f:**

**List of jointly hypermethylated genes in ZAM Amnion and over expressed in ZAM Choriodecidua**

| \| ADAMTS1  ADAMTS5 \| \| --- \| \| ANTXR1 \| \| APOA1 \| \| AR \| \| ATP13A4 \| \| BIN2 \| \| C1orf130 \| \| C1QTNF1 \| \| C2CD4B \| \| C7orf58 \| \| CDA \| \| CEACAM3 \| \| COL14A1 \| \| CORO2A \| \| CXCR6 \| \| CYP11A1 \| \| DENND2A \| \| DIO2 \| \| DSCR8 \| \| EEPD1 \| \| ENTPD8 \| \| FAM153B \| \| FHOD3 \| \| FIGNL2 \| \| FLJ43663 \| \| GCM1 \| \| GDF15 \| \| GH1 \| \| GKN1 \| \| GPC4 \| \| GTSF1 \| \| GYLTL1B \| \| HMGB3 \| \| HN1 \| | \| HPCAL1 \| \| --- \| \| HPGD \| \| HSD3B1 \| \| ICAM2 \| \| IL15 \| \| IL1RL1 \| \| IL20RB \| \| ISM2 \| \| ITGA1 \| \| JAM2 \| \| JAZF1 \| \| KCNJ16 \| \| KIAA1324 \| \| KITLG \| \| KLK3 \| \| LAIR2 \| \| LGALS14 \| \| LIFR \| \| LINGO2 \| \| LIPG \| \| MAGEA8 \| \| MAPT \| \| MCAM \| \| MFSD2A \| \| MMP11 \| \| MMP7 \| \| NFASC \| \| NFE2L3 \| \| NKD2 \| \| NKG7 \| \| NLGN4X \| \| NLRP7 \| \| OGDHL \| \| OLR1 \| | \| OOEP \| \| --- \| \| PAGE4 \| \| PAPPA2 \| \| PAPSS2 \| \| PARD6G \| \| PDCD1LG2 \| \| PLEKHH2 \| \| PPAP2A \| \| PPAPDC3 \| \| PPARG \| \| PPP4R4 \| \| PRB3 \| \| PRG2 \| \| PROK1 \| \| PTGER3 \| \| SCARB1 \| \| SERPINB11 \| \| SLC44A5 \| \| SLC6A2 \| \| SOAT1 \| \| SOHLH2 \| \| SSH2 \| \| STAB2 \| \| SV2B \| \| TLR4 \| \| TM4SF5 \| \| TMEM132B \| \| TNFSF10 \| \| TPST1 \| \| TRIM14 \| \| TSKU \| \| TSNARE1 \| \| TSPAN8 \| \| VAV3 \| \| XAGE3 \| \| ZFP57 \| |
| --- | --- | --- | --- | --- | --- | --- | --- | --- | --- | --- | --- | --- | --- | --- | --- | --- | --- | --- | --- | --- | --- | --- | --- | --- | --- | --- | --- | --- | --- | --- | --- | --- | --- | --- | --- | --- | --- | --- | --- | --- | --- | --- | --- | --- | --- | --- | --- | --- | --- | --- | --- | --- | --- | --- | --- | --- | --- | --- | --- | --- | --- | --- | --- | --- | --- | --- | --- | --- | --- | --- | --- | --- | --- | --- | --- | --- | --- | --- | --- | --- | --- | --- | --- | --- | --- | --- | --- | --- | --- | --- | --- | --- | --- | --- | --- | --- | --- | --- | --- | --- | --- | --- | --- | --- | --- | --- |

**Supplementary file 1g:**

**List of disease mesh-term associated with hypermethylated genes in ZAM Amnion and over expressed in ZAM Choriodecidua**

| \| **MeSH-Term** \| **MeSH-Term id(s)** \| **p-value** \| \| --- \| --- \| --- \| \| Gestational Trophoblastic Disease \| C04.557.465.955.416, C04.850.908.416, C13.703.720.949.416 \| 1,92E-08 \| \| Trophoblastic Neoplasms \| C04.850.908, C13.703.720.949 \| 2,33E-08 \| \| Trophoblastic Neoplasms \| C04.557.465.955 \| 2,35E-08 \| \| Rheumatoid Nodule \| C17.300.775.099.683, C05.799.114.683, C05.550.114.154.683 \| 4,13E-08 \| \| Pregnancy Complications, Neoplastic \| C04.850, C13.703.720 \| 5,56E-08 \| \| Hydatidiform Mole \| C13.703.720.949.416.875, C04.557.465.955.416.812, C04.850.908.416.750 \| 1,73E-07 \| \| Choriocarcinoma \| C04.557.465.955.416.202, C13.703.720.949.416.218, C04.850.908.416.186 \| 1,17E-06 \| \| Choriocarcinoma \| C04.557.470.200.025.455, C04.557.465.955.207 \| 1,19E-06 \| \| Communicable Diseases \| C01.539.221 \| 1,92E-06 \| \| Pre-Eclampsia \| C13.703.395.249 \| 6,78E-06 \| \| Pregnancy, Ectopic \| C13.703.733 \| 9,04E-06 \| \| Hypertension, Pregnancy-Induced \| C13.703.395 \| 9,34E-06 \| \| Metaplasia \| C23.550.589 \| 9,39E-06 \| \| Melanoma, Experimental \| C04.557.465.625.650.510.525, C04.619.600, C04.557.580.625.650.510.525, C04.557.665.510.525 \| 9,57E-06 \| \| Hypersensitivity, Delayed \| C20.543.418 \| 1,09E-05 \| \| Uveitis \| C11.941.879 \| 1,68E-05 \| \| Neovascularization, Pathologic \| C23.550.589.500 \| 2,39E-05 \| \| Rheumatic Diseases \| C05.799 \| 2,61E-05 \| \| Placenta Diseases \| C13.703.590 \| 3,04E-05 \| \| Tendon Injuries \| C26.874 \| 4,43E-05 \| \| Osteoarthritis, Knee \| C05.799.613.500, C05.550.114.606.500 \| 6,64E-05 \| \| Hepatitis, Autoimmune \| C06.552.380.350.050, C20.111.567 \| 6,98E-05 \| \| Dermatitis, Contact \| C17.800.815.255, C17.800.174.255 \| 7,84E-05 \| \| Infertility \| C13.351.500.365 \| 9,15E-05 \| \| Fetal Growth Retardation \| C23.550.393.450, C13.703.277.370, C16.300.390 \| 9,71E-05 \| \| Uveitis, Anterior \| C11.941.879.780.880 \| 1,26E-04 \| \| Sarcoidosis, Pulmonary \| C15.604.515.827.725, C08.381.483.725 \| 1,28E-04 \| \| Infertility, Female \| C13.351.500.365.700 \| 1,34E-04 \| \| Leukemia-Lymphoma, Adult T-Cell \| C15.604.515.560.575.100, C04.557.337.428.580.100, C20.683.515.528.582.100 \| 1,34E-04 \| \| Pregnancy Complications \| C13.703 \| 1,46E-04 \| \| Rhinitis \| C08.730.674 \| 1,56E-04 \| \| Joint Diseases \| C05.550 \| 1,60E-04 \| \| Tuberculosis, Pleural \| C08.730.912, C08.528.928, C01.252.410.040.552.846.877 \| 1,66E-04 \| \| Lichen Planus \| C17.800.859.475.560 \| 1,68E-04 \| \| Panuveitis \| C11.941.879.780 \| 1,86E-04 \| \| Sarcoidosis \| C15.604.515.827 \| 1,89E-04 \| \| Neoplasms, Germ Cell and Embryonal \| C04.557.465 \| 2,01E-04 \| \| Brain Neoplasms \| C04.588.614.250.195, C10.228.140.211, C10.551.240.250 \| 2,05E-04 \| \| Dermatitis, Allergic Contact \| C17.800.815.255.100, C17.800.174.255.100, C20.543.418.150 \| 2,21E-04 \| \| Myocarditis \| C14.280.238.625 \| 2,49E-04 \| \| Leukemia, T-Cell \| C04.557.337.428.580, C20.683.515.528.582, C15.604.515.560.575 \| 2,52E-04 \| \| Alveolar Bone Loss \| C07.465.714.354.500, C05.116.264.150 \| 2,56E-04 \| \| Otitis Media with Effusion \| C09.218.705.663.683 \| 2,74E-04 \| \| Encephalitis, Viral \| C10.228.228.210.150.300, C10.228.228.245.340, C02.182.500.300, C02.290 \| 2,92E-04 \| \| Granuloma \| C15.604.515.292 \| 2,94E-04 \| \| Arteritis \| C14.907.184, C14.907.940.090 \| 2,98E-04 \| \| Temporomandibular Joint Disorders \| C05.550.905, C05.500.607.221.897, C07.678, C07.320.610.291.897, C05.651.243.897 \| 3,01E-04 \| \| Craniomandibular Disorders \| C05.500.607.221, C07.320.610.291, C05.651.243 \| 3,19E-04 \| \| Skin Diseases, Vascular \| C17.800.862 \| 3,30E-04 \| \| Lichenoid Eruptions \| C17.800.859.475 \| 3,36E-04 \| \| Colitis \| C06.405.205.265, C06.405.469.158.188 \| 3,45E-04 \| \| Carcinoma, Merkel Cell \| C04.925.216, C02.928.216, C04.557.465.625.650.240.325, C02.256.721.150, C04.557.580.625.650.240.325, C04.557.470.200.025.370.325 \| 3,49E-04 \| \| Pleurisy \| C08.730.582, C08.528.735 \| 3,49E-04 \| \| Hemostatic Disorders \| C14.907.454 \| 3,54E-04 \| \| Giant Cell Arteritis \| C14.907.940.090.530, C10.228.140.300.850.500, C14.907.184.438, C14.907.940.907.700, C10.114.875.700, C17.800.862.252, C14.907.253.946.700, C20.111.258.962.800 \| 3,59E-04 \| \| Arthritis, Rheumatoid \| C20.111.199 \| 3,60E-04 \| \| Arthritis, Rheumatoid \| C17.300.775.099, C05.799.114, C05.550.114.154 \| 3,61E-04 \| \| Liposarcoma \| C04.557.450.795.465, C04.557.450.550.420 \| 3,66E-04 \| \| Arthritis \| C05.550.114 \| 3,75E-04 \| \| Shock, Hemorrhagic \| C23.550.835.650, C23.550.414.980 \| 3,80E-04 \| \| Demyelinating Autoimmune Diseases, CNS \| C10.314.350 \| 3,87E-04 \| \| Central Nervous System Neoplasms \| C04.588.614.250, C10.551.240 \| 4,01E-04 \| \| Hemostatic Disorders \| C15.378.463.515 \| 4,17E-04 \| \| Female Urogenital Diseases \| C13.351 \| 4,35E-04 \| \| Neoplasms, Glandular and Epithelial \| C04.557.470 \| 4,40E-04 \| \| Respiration Disorders \| C08.618 \| 4,49E-04 \| \| Encephalitozoonosis \| C01.703.617.300 \| 4,90E-04 \| \| Pituitary Neoplasms \| C04.588.614.250.195.885.500.600, C10.551.240.250.700.500.500, C10.228.140.211.885.500.600, C10.228.140.617.477.600 \| 4,97E-04 \| \| Scleroderma, Systemic \| C17.800.784, C17.300.799 \| 4,97E-04 \| \| Multiple Sclerosis, Relapsing-Remitting \| C20.111.258.250.500.600, C10.114.375.500.600, C10.314.350.500.600 \| 4,99E-04 \| \| Arbovirus Infections \| C02.081 \| 5,02E-04 \| \| Hemorrhagic Disorders \| C15.378.463 \| 5,15E-04 \| \| Nervous System Neoplasms \| C10.551 \| 5,26E-04 \| \| Multiple Sclerosis \| C20.111.258.250.500, C10.114.375.500, C10.314.350.500 \| 5,26E-04 \| \| Nervous System Neoplasms \| C04.588.614 \| 5,32E-04 \| \| Rheumatic Diseases \| C17.300.775 \| 5,33E-04 \| \| Hypothalamic Neoplasms \| C10.228.140.211.885.500, C04.588.614.250.195.885.500, C10.228.140.617.477, C10.551.240.250.700.500 \| 5,35E-04 \| \| Plaque, Atherosclerotic \| C23.300.823 \| 5,43E-04 \| \| Conjunctivitis \| C11.187.183 \| 5,54E-04 \| \| Cardiomyopathy, Dilated \| C14.280.238.070, C14.280.195.160 \| 5,55E-04 \| \| Periodontal Atrophy \| C07.465.714.354 \| 5,78E-04 \| \| Lichen Planus, Oral \| C07.465.397, C17.800.859.475.560.397 \| 5,90E-04 \| \| Bone Resorption \| C05.116.264 \| 5,91E-04 \| \| Demyelinating Autoimmune Diseases, CNS \| C10.114.375, C20.111.258.250 \| 6,38E-04 \| \| Rotavirus Infections \| C02.782.791.814 \| 6,51E-04 \| \| Pituitary Neoplasms \| C19.344.609, C19.700.734, C10.228.140.617.738.675, C04.588.322.609 \| 6,70E-04 \| \| Supratentorial Neoplasms \| C04.588.614.250.195.885, C10.228.140.211.885, C10.551.240.250.700 \| 6,73E-04 \| \| Acute Coronary Syndrome \| C14.280.647.124, C23.888.646.215.500.074, C14.907.585.187.074, C14.907.585.124, C14.280.647.187.074 \| 6,77E-04 \| \| Thymus Neoplasms \| C15.604.861, C04.588.894.949 \| 6,78E-04 \| \| Collagen Diseases \| C17.300.200 \| 7,04E-04 \| \| Hypertension \| C14.907.489 \| 7,12E-04 \| \| Polymyositis \| C05.651.594.819, C10.668.491.562.575 \| 7,17E-04 \| \| Crohn Disease \| C06.405.469.432.500, C06.405.205.731.500 \| 7,24E-04 \| \| Dysentery \| C06.405.205.331, C06.405.469.300 \| 7,34E-04 \| \| Adenoma \| C04.557.470.035 \| 7,43E-04 \| \| Bacteroidaceae Infections \| C01.252.400.110 \| 7,43E-04 \| \| Aspergillosis, Allergic Bronchopulmonary \| C17.800.838.208.416.249.074, C01.703.295.328.249.074, C08.674.060, C20.543.480.680.085, C08.730.435.090, C01.703.534.090, C01.539.800.200.383.249.074, C08.381.472.850.500, C01.703.513.249.074 \| 7,50E-04 \| \| Onchocerciasis, Ocular \| C03.300.562, C03.335.508.700.750.361.699.500, C11.294.725.562 \| 7,55E-04 \| \| Eosinophilia \| C15.378.553.231 \| 7,66E-04 \| \| Hypertrophy, Left Ventricular \| C14.280.195.400, C23.300.775.250.400 \| 7,85E-04 \| \| Genital Neoplasms, Female \| C13.351.937.418 \| 7,96E-04 \| \| Mucopolysaccharidosis VI \| C18.452.648.595.600.670, C16.320.565.595.600.670, C16.320.565.202.715.670, C18.452.648.202.715.670, C17.300.550.575.670 \| 8,05E-04 \| \| Esophageal Neoplasms \| C06.405.249.205, C04.588.443.353, C06.301.371.205, C06.405.117.430, C04.588.274.476.205 \| 9,12E-04 \| \| Genital Diseases, Female \| C13.351.500 \| 9,88E-04 \| \| Hepatitis, Chronic \| C06.552.380.350 \| 1,00E-03 \| \| Prolactinoma \| C10.228.140.617.738.675.800, C04.557.470.035.625, C19.700.734.792, C19.344.609.792, C04.588.322.609.792 \| 1,01E-03 \| \| Rupture \| C26.761 \| 1,02E-03 \| \| Encephalitis \| C02.182.500, C10.228.228.210.150 \| 1,02E-03 \| \| Thyroid Diseases \| C19.874 \| 1,02E-03 \| \| Sinusitis \| C09.603.692.752, C08.730.749, C08.460.692.752 \| 1,03E-03 \| \| Uterine Neoplasms \| C13.351.500.852.762 \| 1,05E-03 \| \| Shock, Traumatic \| C26.797 \| 1,07E-03 \| \| Mastocytoma \| C04.557.450.565.465.249, C17.800.508.236 \| 1,07E-03 \| \| Uveal Diseases \| C11.941 \| 1,08E-03 \| \| Vasculitis, Central Nervous System \| C20.111.258.962 \| 1,09E-03 \| \| Encephalomyelitis, Autoimmune, Experimental \| C20.111.258.625.300, C10.314.350.250, C10.228.140.695.562.250, C10.114.703.300 \| 1,10E-03 \| \| Slow Virus Diseases \| C02.839 \| 1,10E-03 \| \| Vasculitis, Central Nervous System \| C14.907.253.946, C10.228.140.300.850, C10.114.875, C14.907.940.907 \| 1,14E-03 \| \| Spondylarthritis \| C05.116.900.853.625, C05.550.114.865 \| 1,15E-03 \| \| Uterine Neoplasms \| C04.588.945.418.948 \| 1,16E-03 \| \| Uterine Neoplasms \| C13.351.937.418.875 \| 1,18E-03 \| \| Venous Thrombosis \| C14.907.355.830.925 \| 1,18E-03 \| \| Connective Tissue Diseases \| C17.300 \| 1,19E-03 \| \| Corneal Neovascularization \| C11.204.290 \| 1,21E-03 \| \| Glomerulonephritis, IGA \| C20.111.525, C12.777.419.570.363.608, C13.351.968.419.570.363.608 \| 1,22E-03 \| \| Cysticercosis \| C03.335.190.902.185 \| 1,28E-03 \| \| Anoxia \| C23.888.852.079 \| 1,29E-03 \| \| Digestive System Abnormalities \| C06.198 \| 1,30E-03 \| \| Autoimmune Diseases of the Nervous System \| C20.111.258, C10.114 \| 1,30E-03 \| \| Nematode Infections \| C03.335.508 \| 1,31E-03 \| \| Acquired Immunodeficiency Syndrome \| C20.673.480.040, C02.839.040, C02.782.815.616.400.040, C02.800.801.400.040 \| 1,33E-03 \| \| Spondylitis \| C05.116.900.853 \| 1,33E-03 \| \| Ascites \| C23.550.081 \| 1,34E-03 \| \| Eye Infections \| C11.294 \| 1,35E-03 \| \| Nervous System Autoimmune Disease, Experimental \| C10.114.703, C20.111.258.625 \| 1,37E-03 \| \| Bone Marrow Neoplasms \| C15.378.400.200, C04.588.448.200, C15.378.190.250 \| 1,38E-03 \| \| Encephalitis \| C10.228.228.245 \| 1,39E-03 \| \| Osteoarthritis, Hip \| C05.799.613.400, C05.550.114.606.400 \| 1,39E-03 \| \| Paracoccidioidomycosis \| C01.703.700, C17.800.838.208.600, C01.703.295.600, C01.539.800.200.600 \| 1,40E-03 \| \| Demyelinating Diseases \| C10.314 \| 1,43E-03 \| \| Growth Disorders \| C23.550.393 \| 1,43E-03 \| \| Spinal Diseases \| C05.116.900 \| 1,43E-03 \| \| Keratitis \| C11.204.564 \| 1,45E-03 \| \| Demyelinating Autoimmune Diseases, CNS \| C10.228.140.695.562 \| 1,48E-03 \| \| Suppuration \| C01.539.830 \| 1,48E-03 \| \| Uterine Diseases \| C13.351.500.852 \| 1,49E-03 \| \| Genital Neoplasms, Female \| C04.588.945.418 \| 1,50E-03 \| \| Cicatrix \| C23.550.355.274 \| 1,54E-03 \| \| Female Urogenital Diseases and Pregnancy Complications \| C13 \| 1,54E-03 \| \| Melanoma \| C04.557.465.625.650.510, C04.557.580.625.650.510, C04.557.665.510 \| 1,54E-03 \| \| Bronchitis \| C08.127.446, C08.381.495.146 \| 1,54E-03 \| \| Nevi and Melanomas \| C04.557.665 \| 1,72E-03 \| \| Ehrlichiosis \| C01.252.400.825.200 \| 1,73E-03 \| \| Neuroendocrine Tumors \| C04.557.580.625.650 \| 1,74E-03 \| \| Nasal Polyps \| C09.603.557, C08.460.572, C23.300.825.557 \| 1,75E-03 \| \| Gastroenteritis \| C06.405.205 \| 1,77E-03 \| \| Sarcopenia \| C10.597.613.612.500, C23.888.592.608.612.500, C23.300.070.500.500 \| 1,78E-03 \| \| Neuroendocrine Tumors \| C04.557.465.625.650 \| 1,79E-03 \| \| Abdominal Neoplasms \| C04.588.033 \| 1,81E-03 \| \| Wallerian Degeneration \| C23.550.737.750 \| 1,85E-03 \| \| Flavivirus Infections \| C02.782.350.250 \| 1,87E-03 \| \| Endometrial Neoplasms \| C13.351.500.852.762.200 \| 1,88E-03 \| \| Dermatitis \| C17.800.174 \| 1,91E-03 \| \| Rhinitis \| C08.460.799, C09.603.799 \| 1,92E-03 \| \| Neoplasms, Adipose Tissue \| C04.557.450.550 \| 1,93E-03 \| \| Ehrlichiosis \| C01.252.400.054.160 \| 1,98E-03 \| \| Liver Cirrhosis, Biliary \| C06.552.630.400, C06.552.150.250, C06.130.120.135.250.250 \| 1,99E-03 \| \| Shock \| C23.550.835 \| 2,00E-03 \| \| Neoplasms, Connective and Soft Tissue \| C04.557.450 \| 2,02E-03 \| \| Microsporidiosis \| C01.703.617 \| 2,04E-03 \| \| Barrett Esophagus \| C06.405.117.102, C06.198.102 \| 2,10E-03 \| \| Musculoskeletal Diseases \| C05 \| 2,11E-03 \| \| Hypereosinophilic Syndrome \| C15.378.553.231.549 \| 2,12E-03 \| \| Sarcoma \| C04.557.450.795 \| 2,15E-03 \| \| Corneal Diseases \| C11.204 \| 2,15E-03 \| \| Cardiomegaly \| C14.280.195 \| 2,16E-03 \| \| Tendinopathy \| C26.874.800 \| 2,16E-03 \| \| Biliary Tract Diseases \| C06.130 \| 2,19E-03 \| \| Brain Abscess \| C01.323, C01.539.830.025.160, C10.228.140.116, C10.228.228.090 \| 2,19E-03 \| \| Eye Infections \| C01.539.375 \| 2,22E-03 \| \| AIDS-Related Opportunistic Infections \| C20.673.480.100, C01.539.597.050, C03.684.050, C02.597.050, C02.782.815.616.400.100 \| 2,22E-03 \| \| Reoviridae Infections \| C02.782.791 \| 2,25E-03 \| \| Periodontal Diseases \| C07.465.714 \| 2,27E-03 \| \| Urogenital Neoplasms \| C13.351.937 \| 2,29E-03 \| \| Pulmonary Aspergillosis \| C08.381.472.850 \| 2,29E-03 \| \| Herpes Genitalis \| C13.351.500.342, C02.800.801.350, C12.294.329, C02.256.466.382.290 \| 2,32E-03 \| \| Prosthesis Failure \| C23.550.767.865 \| 2,33E-03 \| \| Cardiomegaly \| C23.300.775.250 \| 2,36E-03 \| \| Epiretinal Membrane \| C11.768.328 \| 2,39E-03 \| \| Osteoarthritis \| C05.799.613, C05.550.114.606 \| 2,43E-03 \| \| Adenocarcinoma \| C04.557.470.200.025 \| 2,45E-03 \| \| Bronchial Hyperreactivity \| C08.127.210 \| 2,48E-03 \| \| Endometrial Neoplasms \| C04.588.945.418.948.585 \| 2,50E-03 \| \| Pituitary Diseases \| C10.228.140.617.738 \| 2,51E-03 \| \| Leishmaniasis, Diffuse Cutaneous \| C03.858.560.400.350, C17.800.838.775.560.400.350, C03.752.300.500.400.350 \| 2,54E-03 \| \| Taeniasis \| C03.335.190.902 \| 2,56E-03 \| \| Xanthomatosis \| C18.452.584.750 \| 2,56E-03 \| \| Abdominal Pain \| C23.888.646.100, C23.888.821.030 \| 2,57E-03 \| \| Rhabdoviridae Infections \| C02.782.580.830 \| 2,59E-03 \| \| Endometrial Neoplasms \| C13.351.937.418.875.200 \| 2,60E-03 \| \| Neoplasm Metastasis \| C23.550.727.650, C04.697.650 \| 2,61E-03 \| \| Dry Eye Syndromes \| C11.496.260 \| 2,61E-03 \| \| Disease Resistance \| C23.550.291.671 \| 2,61E-03 \| \| Kashin-Beck Disease \| C05.116.099.708.534 \| 2,63E-03 \| \| Tendinopathy \| C05.651.869 \| 2,66E-03 \| \| Placenta Accreta \| C13.703.420.643, C13.703.590.609 \| 2,72E-03 \| \| Skin Diseases, Eczematous \| C17.800.815 \| 2,73E-03 \| \| Aggressive Periodontitis \| C07.465.714.533.161 \| 2,77E-03 \| \| Pyometra \| C13.351.500.852.544 \| 2,81E-03 \| \| Anaplasmataceae Infections \| C01.252.400.054 \| 2,84E-03 \| \| Periapical Periodontitis \| C07.465.714.306.700, C07.465.714.533.487, C07.320.830.700 \| 2,88E-03 \| \| Vasculitis \| C14.907.940 \| 2,90E-03 \| \| Mononegavirales Infections \| C02.782.580 \| 2,94E-03 \| \| Multiple Myeloma \| C15.378.463.515.460, C14.907.454.460, C20.683.780.650, C15.378.147.780.650 \| 2,97E-03 \| \| Trophoblastic Tumor, Placental Site \| C04.850.908.416.186.875, C04.557.465.955.207.875, C04.557.465.955.416.202.875, C13.703.720.949.416.218.875, C04.557.470.200.025.455.875 \| 3,00E-03 \| \| Graves Ophthalmopathy \| C11.270.842, C20.111.555.500, C19.874.397.370.500, C19.874.283.605.500, C11.675.349.500.500 \| 3,03E-03 \| \| Obstetric Labor Complications \| C13.703.420 \| 3,06E-03 \| \| Shwartzman Phenomenon \| C15.378.463.515.810, C14.907.454.810, C14.907.940.890 \| 3,09E-03 \| \| Mandibular Diseases \| C05.500.607 \| 3,11E-03 \| \| Multiple Myeloma \| C04.557.595.500, C20.683.515.845 \| 3,12E-03 \| \| HELLP Syndrome \| C13.703.395.186 \| 3,14E-03 \| \| Dermatomyositis \| C17.800.185, C10.668.491.562.575.500, C17.300.250, C05.651.594.819.500, C05.651.594.297, C10.668.491.562.150 \| 3,18E-03 \| \| Parapsoriasis \| C17.800.859.575 \| 3,19E-03 \| \| Neoplasms, Connective Tissue \| C04.557.450.565 \| 3,23E-03 \| \| Orthomyxoviridae Infections \| C02.782.620 \| 3,23E-03 \| \| Salivary Gland Diseases \| C07.465.815 \| 3,26E-03 \| \| Secernentea Infections \| C03.335.508.700 \| 3,27E-03 \| \| Fetal Diseases \| C16.300 \| 3,30E-03 \| \| Paranasal Sinus Diseases \| C08.460.692, C09.603.692 \| 3,31E-03 \| \| Bile Duct Diseases \| C06.130.120 \| 3,31E-03 \| \| Neoplasms, Vascular Tissue \| C04.557.645 \| 3,32E-03 \| \| Esophageal Diseases \| C06.405.117 \| 3,32E-03 \| \| Alveolitis, Extrinsic Allergic \| C08.381.483.125, C20.543.480.680.075, C08.674.055 \| 3,34E-03 \| \| Graves Disease \| C19.874.283.605, C19.874.397.370, C11.675.349.500, C20.111.555 \| 3,41E-03 \| \| Neoplasms by Site \| C04.588 \| 3,48E-03 \| \| Meningitis, Bacterial \| C10.228.228.507.280, C01.252.200.500, C10.228.228.180.500 \| 3,50E-03 \| \| Fetal Diseases \| C13.703.277 \| 3,53E-03 \| \| Thrombosis \| C14.907.355.830 \| 3,54E-03 \| \| Central Nervous System Viral Diseases \| C02.182, C10.228.228.210 \| 3,55E-03 \| \| Lacrimal Apparatus Diseases \| C11.496 \| 3,55E-03 \| \| Mandibular Diseases \| C07.320.610 \| 3,56E-03 \| \| Head and Neck Neoplasms \| C04.588.443 \| 3,57E-03 \| \| Fibrosis \| C23.550.355 \| 3,58E-03 \| \| Angiolymphoid Hyperplasia with Eosinophilia \| C17.800.060, C15.604.515.292.007, C15.378.553.231.085 \| 3,59E-03 \| \| Respiratory Syncytial Virus Infections \| C02.782.580.600.620.750 \| 3,61E-03 \| \| Pituitary Diseases \| C19.700 \| 3,65E-03 \| \| Pulmonary Emphysema \| C08.381.495.389.750 \| 3,66E-03 \| \| Spinal Cord Compression \| C26.819.678, C10.228.854.761 \| 3,72E-03 \| \| Abscess \| C01.539.830.025 \| 3,74E-03 \| \| Otitis Media \| C09.218.705.663 \| 3,74E-03 \| \| Helminthiasis \| C03.335 \| 3,74E-03 \| \| Osteolysis \| C05.116.264.579 \| 3,79E-03 \| \| Testicular Hydrocele \| C12.294.882 \| 3,80E-03 \| \| Pneumovirus Infections \| C02.782.580.600.620 \| 3,82E-03 \| \| Granuloma \| C23.550.382 \| 3,84E-03 \| \| Pain \| C23.888.646 \| 3,84E-03 \| \| Arthritis, Psoriatic \| C17.800.859.675.175, C05.550.114.865.800.424, C05.116.900.853.625.800.424, C05.550.114.145 \| 3,85E-03 \| \| Abortion, Habitual \| C13.703.039.089 \| 3,85E-03 \| \| Pneumonia, Pneumocystis \| C08.730.610.675, C08.730.435.700, C01.703.770.700, C01.703.534.700, C08.381.472.700, C08.381.677.675 \| 3,86E-03 \| \| Blood Loss, Surgical \| C23.550.505.300, C23.550.414.300 \| 3,90E-03 \| \| Polyradiculoneuropathy \| C20.111.258.750, C10.314.750, C10.114.750 \| 3,93E-03 \| \| Balantidiasis \| C06.405.469.452.146, C03.752.200.146, C03.432.146 \| 3,95E-03 \| \| Signs and Symptoms, Respiratory \| C23.888.852 \| 3,98E-03 \| \| Influenza, Human \| C02.782.620.365, C08.730.310 \| 3,98E-03 \| \| Pancreatitis, Chronic \| C06.689.750.830 \| 3,99E-03 \| \| Strongylida Infections \| C03.335.508.700.775 \| 3,99E-03 \| \| X-Linked Combined Immunodeficiency Diseases \| C20.673.815.500, C16.614.815.500, C16.320.322.968 \| 4,02E-03 \| \| Signs and Symptoms \| C23.888 \| 4,03E-03 \| \| Hypertension, Pregnancy-Induced \| C14.907.489.480 \| 4,04E-03 \| \| Dog Diseases \| C22.268 \| 4,09E-03 \| \| Hypothalamic Diseases \| C10.228.140.617 \| 4,11E-03 \| \| Peritoneal Neoplasms \| C04.588.274.780, C06.844.620, C06.301.780, C04.588.033.513 \| 4,12E-03 \| \| Arthritis, Experimental \| C05.550.114.015 \| 4,12E-03 \| \| Asymptomatic Infections \| C23.550.291.187.500 \| 4,13E-03 \| \| Muscular Dystrophy, Animal \| C22.595 \| 4,13E-03 \| \| Keratitis, Herpetic \| C11.294.800.475, C02.256.466.382.465, C11.204.564.425, C02.325.465 \| 4,22E-03 \| \| Respiratory Insufficiency \| C08.618.846 \| 4,25E-03 \| \| Chagas Cardiomyopathy \| C14.280.238.190, C03.752.300.900.200.190 \| 4,27E-03 \| \| Gingival Overgrowth \| C07.465.714.258.428 \| 4,27E-03 \| \| Pneumonia \| C08.381.677, C08.730.610 \| 4,29E-03 \| \| Polyradiculoneuropathy \| C10.668.829.800.750 \| 4,31E-03 \| \| Bone Neoplasms \| C04.588.149, C05.116.231 \| 4,33E-03 \| \| Tuberculosis, Pulmonary \| C08.381.922, C08.730.939, C01.252.410.040.552.846.899 \| 4,34E-03 \| \| Synovitis \| C05.550.870 \| 4,40E-03 \| \| Paramyxoviridae Infections \| C02.782.580.600 \| 4,45E-03 \| \| Lupus Erythematosus, Cutaneous \| C17.300.475, C17.800.480 \| 4,46E-03 \| \| Pneumocystis Infections \| C01.703.770 \| 4,46E-03 \| \| Necrobiotic Disorders \| C17.800.550, C17.300.200.495 \| 4,47E-03 \| \| Paraproteinemias \| C15.378.147.780 \| 4,48E-03 \| \| Exophthalmos \| C11.675.349 \| 4,49E-03 \| \| Arteriosclerosis \| C14.907.137.126 \| 4,52E-03 \| \| Paraproteinemias \| C20.683.780 \| 4,55E-03 \| \| Stomatitis \| C07.465.864 \| 4,61E-03 \| \| Down Syndrome \| C16.131.260.260, C10.597.606.643.220, C16.131.077.327, C16.320.180.260 \| 4,62E-03 \| \| Lymphatic Metastasis \| C23.550.727.650.560, C04.697.650.560 \| 4,62E-03 \| \| Gastritis \| C06.405.748.398, C06.405.205.697 \| 4,65E-03 \| \| Neoplasms, Plasma Cell \| C04.557.595 \| 4,66E-03 \| \| Ovarian Diseases \| C19.391.630 \| 4,67E-03 \| \| Otitis \| C09.218.705 \| 4,67E-03 \| \| Ovarian Diseases \| C13.351.500.056.630 \| 4,70E-03 \| \| Bronchitis \| C08.730.099 \| 4,80E-03 \| \| Dysentery, Bacillary \| C06.405.205.331.479, C06.405.469.300.479, C01.252.400.310.229 \| 4,81E-03 \| \| Glomerulonephritis \| C13.351.968.419.570.363, C12.777.419.570.363 \| 4,82E-03 \| \| Skin Diseases, Vesiculobullous \| C17.800.865 \| 4,86E-03 \| \| Behcet Syndrome \| C17.800.862.150, C07.465.075, C14.907.940.100, C11.941.879.780.880.200 \| 4,86E-03 \| \| Necrosis \| C23.550.717 \| 4,88E-03 \| \| Systemic Vasculitis \| C14.907.940.897 \| 4,90E-03 \| \| Scrub Typhus \| C01.252.400.780.850 \| 4,94E-03 \| \| Pleural Effusion, Malignant \| C08.528.694.700, C04.588.894.797.640.700, C08.785.640.700, C08.528.652.700 \| 4,96E-03 \| \| Ulcer \| C23.550.891 \| 4,96E-03 \| \| Skin Neoplasms \| C17.800.882, C04.588.805 \| 4,97E-03 \| \| Conjunctival Diseases \| C11.187 \| 5,01E-03 \| \| Epidermolysis Bullosa \| C17.800.865.410, C16.131.831.493, C17.800.804.493, C17.800.827.275 \| 5,02E-03 \| \| Signs and Symptoms, Digestive \| C23.888.821 \| 5,02E-03 \| \| Adnexal Diseases \| C13.351.500.056 \| 5,14E-03 \| \| Meningitis \| C10.228.228.507 \| 5,15E-03 \| \| Polyomavirus Infections \| C02.256.721 \| 5,16E-03 \| \| Cough \| C08.618.248, C23.888.852.293 \| 5,17E-03 \| \| Embolism and Thrombosis \| C14.907.355 \| 5,25E-03 \| \| Rhinitis, Allergic, Perennial \| C08.674.453.500, C20.543.480.680.443.500, C08.460.799.315.500, C09.603.799.315.500 \| 5,26E-03 \| \| Myositis \| C10.668.491.562 \| 5,30E-03 \| \| Angina Pectoris \| C23.888.646.215.500 \| 5,38E-03 \| \| Eye Infections, Bacterial \| C01.539.375.354, C01.252.354, C11.294.354 \| 5,39E-03 \| \| Hip Dislocation, Congenital \| C16.131.621.449, C05.660.449 \| 5,43E-03 \| \| Arthralgia \| C05.550.091, C23.888.646.130 \| 5,44E-03 \| \| Cholestasis \| C06.130.120.135 \| 5,49E-03 \| \| Cholestasis, Intrahepatic \| C06.130.120.135.250, C06.552.150 \| 5,51E-03 \| \| Hematologic Neoplasms \| C04.588.448, C15.378.400 \| 5,68E-03 \| \| Spondylarthropathies \| C05.116.900.853.625.800, C05.550.114.865.800 \| 5,72E-03 \| \| Angina Pectoris \| C14.280.647.187, C14.907.585.187 \| 5,80E-03 \| \| Dermatitis, Seborrheic \| C17.800.794.230, C17.800.174.580, C17.800.859.350, C17.800.815.580 \| 5,81E-03 \| \| Cicatrix, Hypertrophic \| C23.550.355.274.505 \| 5,95E-03 \| \| Herpes Simplex \| C02.256.466.382 \| 6,02E-03 \| \| Neoplasms, Neuroepithelial \| C04.557.470.670, C04.557.580.625.600, C04.557.465.625.600 \| 6,08E-03 \| \| Occupational Diseases \| C24 \| 6,10E-03 \| \| Asthma \| C08.381.495.108 \| 6,10E-03 \| \| Central Nervous System Bacterial Infections \| C01.252.200 \| 6,16E-03 \| \| Myositis \| C05.651.594 \| 6,19E-03 \| \| Cranial Nerve Injuries \| C26.260.237, C10.292.262, C26.915.300.400, C10.900.300.218 \| 6,20E-03 \| \| Tooth Injuries \| C26.900, C07.793.850 \| 6,21E-03 \| \| Asthma \| C20.543.480.680.095 \| 6,27E-03 \| \| Atherosclerosis \| C14.907.137.126.307 \| 6,29E-03 \| \| Central Nervous System Infections \| C10.228.228 \| 6,36E-03 \| \| Sjogren's Syndrome \| C07.465.815.929.669, C05.799.114.774, C05.550.114.154.774, C20.111.199.774, C11.496.260.719, C17.300.775.099.774 \| 6,39E-03 \| \| Central Nervous System Bacterial Infections \| C10.228.228.180 \| 6,43E-03 \| \| Cryptogenic Organizing Pneumonia \| C08.381.483.487.249, C08.381.495.146.135.140.200, C08.127.446.135.140.200 \| 6,48E-03 \| \| Periapical Diseases \| C07.320.830, C07.465.714.306 \| 6,49E-03 \| \| Enteritis \| C06.405.469.326, C06.405.205.462 \| 6,51E-03 \| \| Asthma \| C08.674.095, C08.127.108 \| 6,55E-03 \| \| Urogenital Neoplasms \| C04.588.945 \| 6,55E-03 \| \| Autoimmune Diseases \| C20.111 \| 6,63E-03 \| \| Chondrosarcoma \| C04.557.450.565.280, C04.557.450.795.300 \| 6,70E-03 \| \| Chest Pain \| C23.888.646.215 \| 6,71E-03 \| \| Lyme Neuroborreliosis \| C01.252.400.825.480.700, C01.252.400.155.569.600, C10.228.228.180.437, C01.252.200.450, C01.252.847.193.569.600 \| 6,75E-03 \| \| Obstetric Labor, Premature \| C13.703.420.491 \| 6,83E-03 \| \| Pneumonia, Pneumococcal \| C08.381.677.540.550, C01.252.620.550, C01.252.410.890.670.750, C08.730.610.540.550 \| 6,86E-03 \| \| Male Urogenital Diseases \| C12 \| 6,87E-03 \| \| Arterial Occlusive Diseases \| C14.907.137 \| 6,90E-03 \| \| Conjunctivitis, Allergic \| C11.187.183.200, C20.543.480.200 \| 6,99E-03 \| \| Tick-Borne Diseases \| C01.252.400.825 \| 6,99E-03 \| \| Sarcoma, Kaposi \| C04.557.645.750, C04.557.450.795.850, C02.256.466.860 \| 7,03E-03 \| \| Mastocytosis \| C04.557.450.565.465 \| 7,03E-03 \| \| Neurocysticercosis \| C03.335.190.902.185.550, C03.105.250.550, C10.228.228.205.250.550 \| 7,03E-03 \| \| Hernia \| C23.300.707 \| 7,04E-03 \| \| Odontogenic Cysts \| C07.320.450.670, C05.500.470.690, C04.182.089.530.690 \| 7,05E-03 \| \| Ovarian Neoplasms \| C19.344.410, C04.588.322.455 \| 7,05E-03 \| \| Rhabditida Infections \| C03.335.508.700.700 \| 7,17E-03 \| \| Strongyloidiasis \| C03.335.508.700.700.799 \| 7,17E-03 \| \| Spondylitis, Ankylosing \| C05.116.900.853.625.800.850, C05.550.069.680, C05.550.114.865.800.850 \| 7,20E-03 \| \| Enterocolitis, Necrotizing \| C06.405.205.596.700, C06.405.469.363.700 \| 7,24E-03 \| \| Orbital Diseases \| C11.675 \| 7,26E-03 \| \| Pleural Diseases \| C08.528 \| 7,28E-03 \| \| Animal Diseases \| C22 \| 7,35E-03 \| \| Measles \| C02.782.580.600.500.500 \| 7,44E-03 \| \| Leukoencephalopathies \| C10.228.140.695 \| 7,45E-03 \| \| Bovine Virus Diarrhea-Mucosal Disease \| C22.196.106, C02.782.350.675.106 \| 7,46E-03 \| \| Environmental Illness \| C21.223, C20.543.312 \| 7,46E-03 \| \| Pregnancy, Tubal \| C13.703.733.703 \| 7,46E-03 \| \| Jaw Cysts \| C05.500.470, C04.182.089.530, C07.320.450 \| 7,58E-03 \| \| Diabetic Angiopathies \| C14.907.320, C19.246.099.500 \| 7,59E-03 \| \| Trachoma \| C01.539.375.354.220.800, C01.252.354.225.800, C11.294.354.220.800, C01.252.400.210.210.800, C11.187.183.220.889, C11.204.813 \| 7,61E-03 \| \| Lymphocytic Choriomeningitis \| C10.228.228.507.700.500, C02.587.580, C02.182.550.500, C10.228.228.210.500.500, C02.782.082.580 \| 7,64E-03 \| \| Pulmonary Eosinophilia \| C08.381.750, C15.378.553.231.549.750 \| 7,71E-03 \| \| Acute Pain \| C10.597.617.088, C23.888.646.115 \| 7,76E-03 \| \| Sleep-Wake Transition Disorders \| C10.886.659.700 \| 7,88E-03 \| \| Entropion \| C11.338.443 \| 7,88E-03 \| \| Pouchitis \| C06.405.469.420.520.500, C06.405.469.326.875.500, C06.405.205.462.624.500 \| 7,91E-03 \| \| Colic \| C23.888.821.030.500, C23.888.646.100.600 \| 7,91E-03 \| \| Xerostomia \| C07.465.815.929 \| 7,91E-03 \| \| Diabetic Retinopathy \| C14.907.320.382, C19.246.099.500.382, C11.768.257 \| 7,98E-03 \| \| Heart Valve Diseases \| C14.280.484 \| 8,02E-03 \| \| Ovarian Neoplasms \| C13.351.937.418.685, C19.391.630.705, C13.351.500.056.630.705 \| 8,04E-03 \| \| Dyspnea \| C08.618.326, C23.888.852.371 \| 8,12E-03 \| \| Bronchiolitis Obliterans \| C08.127.446.135.140, C08.381.495.146.135.140 \| 8,33E-03 \| \| Hypertrophy \| C23.300.775 \| 8,37E-03 \| \| Malaria, Cerebral \| C03.105.300.500, C10.228.228.205.300.500, C03.752.530.620, C03.752.530.650.675 \| 8,48E-03 \| \| Hookworm Infections \| C03.335.508.700.775.455 \| 8,51E-03 \| \| Abortion, Spontaneous \| C13.703.039 \| 8,56E-03 \| \| Blood Protein Disorders \| C15.378.147 \| 8,61E-03 \| \| Picornaviridae Infections \| C02.782.687 \| 8,71E-03 \| \| Muscular Dystrophy, Duchenne \| C16.320.577.300, C05.651.534.500.300, C10.668.491.175.500.300, C16.320.322.562 \| 8,72E-03 \| \| Retinitis \| C11.768.773 \| 8,77E-03 \| \| Opportunistic Infections \| C01.539.597, C02.597, C03.684 \| 8,82E-03 \| \| Dermatomycoses \| C17.800.838.208, C01.539.800.200, C01.703.295 \| 8,82E-03 \| \| Hernia, Abdominal \| C23.300.707.374 \| 8,84E-03 \| \| Carcinoma, Renal Cell \| C12.758.820.750.160, C12.777.419.473.160, C13.351.937.820.535.160, C04.557.470.200.025.390, C13.351.968.419.473.160, C04.588.945.947.535.160 \| 8,85E-03 \| \| Ankylosis \| C05.550.069 \| 8,86E-03 \| \| Leukemia, Lymphoid \| C04.557.337.428, C20.683.515.528, C15.604.515.560 \| 8,92E-03 \| \| Bronchiolitis, Viral \| C02.109, C08.127.446.135.321, C08.381.495.146.135.321, C08.730.099.135.321 \| 8,98E-03 \| \| Toxoplasmosis, Animal \| C03.701.688.817, C22.674.710.817, C03.752.625.817, C03.752.250.800.110 \| 9,06E-03 \| \| Thoracic Neoplasms \| C04.588.894 \| 9,23E-03 \| \| Intellectual Disability \| C10.597.606.643 \| 9,26E-03 \| \| Liver Cirrhosis \| C06.552.630 \| 9,28E-03 \| \| RNA Virus Infections \| C02.782 \| 9,29E-03 \| \| Superinfection \| C03.684.880, C01.539.597.880, C02.597.880 \| 9,30E-03 \| \| Disorders of Environmental Origin \| C21 \| 9,30E-03 \| \| Prostatic Neoplasms \| C04.588.945.440.770, C12.294.260.750, C12.294.565.625, C12.758.409.750 \| 9,31E-03 \| \| Pneumococcal Infections \| C01.252.410.890.670 \| 9,36E-03 \| \| Neoplasms by Histologic Type \| C04.557 \| 9,43E-03 \| \| Eye Infections, Viral \| C11.294.800, C02.325 \| 9,44E-03 \| \| Fractures, Bone \| C26.404 \| 9,46E-03 \| \| Subacute Sclerosing Panencephalitis \| C10.228.228.210.150.300.600, C02.290.700, C10.228.228.245.340.700, C02.782.580.600.500.500.800, C02.182.500.300.600, C02.839.862 \| 9,46E-03 \| \| Central Nervous System Helminthiasis \| C10.228.228.205.250, C03.105.250 \| 9,46E-03 \| \| Diabetes Mellitus \| C19.246 \| 9,50E-03 \| \| Inflammatory Bowel Diseases \| C06.405.469.432, C06.405.205.731 \| 9,56E-03 \| \| Purpura \| C15.378.100.802, C23.888.885.687, C23.550.414.950 \| 9,71E-03 \| \| Respiratory Tract Neoplasms \| C08.785 \| 9,80E-03 \| \| Intervertebral Disc Displacement \| C23.300.707.952, C05.116.900.307 \| 9,83E-03 \| \| Lyme Disease \| C01.252.400.825.480, C01.252.400.155.569, C01.252.847.193.569 \| 9,91E-03 \| |
| --- | --- | --- | --- | --- | --- | --- | --- | --- | --- | --- | --- | --- | --- | --- | --- | --- | --- | --- | --- | --- | --- | --- | --- | --- | --- | --- | --- | --- | --- | --- | --- | --- | --- | --- | --- | --- | --- | --- | --- | --- | --- | --- | --- | --- | --- | --- | --- | --- | --- | --- | --- | --- | --- | --- | --- | --- | --- | --- | --- | --- | --- | --- | --- | --- | --- | --- | --- | --- | --- | --- | --- | --- | --- | --- | --- | --- | --- | --- | --- | --- | --- | --- | --- | --- | --- | --- | --- | --- | --- | --- | --- | --- | --- | --- | --- | --- | --- | --- | --- | --- | --- | --- | --- | --- | --- | --- | --- | --- | --- | --- | --- | --- | --- | --- | --- | --- | --- | --- | --- | --- | --- | --- | --- | --- | --- | --- | --- | --- | --- | --- | --- | --- | --- | --- | --- | --- | --- | --- | --- | --- | --- | --- | --- | --- | --- | --- | --- | --- | --- | --- | --- | --- | --- | --- | --- | --- | --- | --- | --- | --- | --- | --- | --- | --- | --- | --- | --- | --- | --- | --- | --- | --- | --- | --- | --- | --- | --- | --- | --- | --- | --- | --- | --- | --- | --- | --- | --- | --- | --- | --- | --- | --- | --- | --- | --- | --- | --- | --- | --- | --- | --- | --- | --- | --- | --- | --- | --- | --- | --- | --- | --- | --- | --- | --- | --- | --- | --- | --- | --- | --- | --- | --- | --- | --- | --- | --- | --- | --- | --- | --- | --- | --- | --- | --- | --- | --- | --- | --- | --- | --- | --- | --- | --- | --- | --- | --- | --- | --- | --- | --- | --- | --- | --- | --- | --- | --- | --- | --- | --- | --- | --- | --- | --- | --- | --- | --- | --- | --- | --- | --- | --- | --- | --- | --- | --- | --- | --- | --- | --- | --- | --- | --- | --- | --- | --- | --- | --- | --- | --- | --- | --- | --- | --- | --- | --- | --- | --- | --- | --- | --- | --- | --- | --- | --- | --- | --- | --- | --- | --- | --- | --- | --- | --- | --- | --- | --- | --- | --- | --- | --- | --- | --- | --- | --- | --- | --- | --- | --- | --- | --- | --- | --- | --- | --- | --- | --- | --- | --- | --- | --- | --- | --- | --- | --- | --- | --- | --- | --- | --- | --- | --- | --- | --- | --- | --- | --- | --- | --- | --- | --- | --- | --- | --- | --- | --- | --- | --- | --- | --- | --- | --- | --- | --- | --- | --- | --- | --- | --- | --- | --- | --- | --- | --- | --- | --- | --- | --- | --- | --- | --- | --- | --- | --- | --- | --- | --- | --- | --- | --- | --- | --- | --- | --- | --- | --- | --- | --- | --- | --- | --- | --- | --- | --- | --- | --- | --- | --- | --- | --- | --- | --- | --- | --- | --- | --- | --- | --- | --- | --- | --- | --- | --- | --- | --- | --- | --- | --- | --- | --- | --- | --- | --- | --- | --- | --- | --- | --- | --- | --- | --- | --- | --- | --- | --- | --- | --- | --- | --- | --- | --- | --- | --- | --- | --- | --- | --- | --- | --- | --- | --- | --- | --- | --- | --- | --- | --- | --- | --- | --- | --- | --- | --- | --- | --- | --- | --- | --- | --- | --- | --- | --- | --- | --- | --- | --- | --- | --- | --- | --- | --- | --- | --- | --- | --- | --- | --- | --- | --- | --- | --- | --- | --- | --- | --- | --- | --- | --- | --- | --- | --- | --- | --- | --- | --- | --- | --- | --- | --- | --- | --- | --- | --- | --- | --- | --- | --- | --- | --- | --- | --- | --- | --- | --- | --- | --- | --- | --- | --- | --- | --- | --- | --- | --- | --- | --- | --- | --- | --- | --- | --- | --- | --- | --- | --- | --- | --- | --- | --- | --- | --- | --- | --- | --- | --- | --- | --- | --- | --- | --- | --- | --- | --- | --- | --- | --- | --- | --- | --- | --- | --- | --- | --- | --- | --- | --- | --- | --- | --- | --- | --- | --- | --- | --- | --- | --- | --- | --- | --- | --- | --- | --- | --- | --- | --- | --- | --- | --- | --- | --- | --- | --- | --- | --- | --- | --- | --- | --- | --- | --- | --- | --- | --- | --- | --- | --- | --- | --- | --- | --- | --- | --- | --- | --- | --- | --- | --- | --- | --- | --- | --- | --- | --- | --- | --- | --- | --- | --- | --- | --- | --- | --- | --- | --- | --- | --- | --- | --- | --- | --- | --- | --- | --- | --- | --- | --- | --- | --- | --- | --- | --- | --- | --- | --- | --- | --- | --- | --- | --- | --- | --- | --- | --- | --- | --- | --- | --- | --- | --- | --- | --- | --- | --- | --- | --- | --- | --- | --- | --- | --- | --- | --- | --- | --- | --- | --- | --- | --- | --- | --- | --- | --- | --- | --- | --- | --- | --- | --- | --- | --- | --- | --- | --- | --- | --- | --- | --- | --- | --- | --- | --- | --- | --- | --- | --- | --- | --- | --- | --- | --- | --- | --- | --- | --- | --- | --- | --- | --- | --- | --- | --- | --- | --- | --- | --- | --- | --- | --- | --- | --- | --- | --- | --- | --- | --- | --- | --- | --- | --- | --- | --- | --- | --- | --- | --- | --- | --- | --- | --- | --- | --- | --- | --- | --- | --- | --- | --- | --- | --- | --- | --- | --- | --- | --- | --- | --- | --- | --- | --- | --- | --- | --- | --- | --- | --- | --- | --- | --- | --- | --- | --- | --- | --- | --- | --- | --- | --- | --- | --- | --- | --- | --- | --- | --- | --- | --- | --- | --- | --- | --- | --- | --- | --- | --- | --- | --- | --- | --- | --- | --- | --- | --- | --- | --- | --- | --- | --- | --- | --- | --- | --- | --- | --- | --- | --- | --- | --- | --- | --- | --- | --- | --- | --- | --- | --- | --- | --- | --- | --- | --- | --- | --- | --- | --- | --- | --- | --- | --- | --- | --- | --- | --- | --- | --- | --- | --- | --- | --- | --- | --- | --- | --- | --- | --- | --- | --- | --- | --- | --- | --- | --- | --- | --- | --- | --- | --- | --- | --- | --- | --- | --- | --- | --- | --- | --- | --- | --- | --- | --- | --- | --- | --- | --- | --- | --- | --- | --- | --- | --- | --- | --- | --- | --- | --- | --- | --- | --- | --- | --- | --- | --- | --- | --- | --- | --- | --- | --- | --- | --- | --- | --- | --- | --- | --- | --- | --- | --- | --- | --- | --- | --- | --- | --- | --- | --- | --- | --- | --- | --- | --- | --- | --- | --- | --- | --- | --- | --- | --- | --- | --- | --- | --- | --- | --- | --- | --- | --- | --- | --- | --- | --- | --- | --- | --- | --- | --- | --- | --- | --- | --- | --- | --- | --- | --- | --- | --- | --- | --- | --- | --- | --- | --- | --- | --- | --- | --- | --- | --- | --- | --- | --- | --- | --- | --- | --- | --- | --- | --- | --- | --- | --- | --- | --- | --- | --- | --- | --- | --- | --- | --- | --- | --- | --- | --- | --- | --- | --- | --- | --- | --- | --- | --- | --- | --- | --- | --- | --- | --- | --- | --- | --- | --- | --- | --- | --- | --- | --- | --- | --- | --- | --- | --- | --- | --- | --- | --- | --- | --- | --- | --- | --- | --- | --- | --- | --- | --- | --- | --- | --- | --- | --- | --- | --- | --- | --- | --- | --- | --- | --- | --- | --- | --- | --- | --- | --- | --- | --- | --- | --- | --- | --- | --- | --- | --- | --- | --- | --- | --- | --- | --- | --- | --- | --- | --- | --- | --- | --- | --- | --- | --- | --- | --- | --- | --- | --- | --- | --- | --- | --- | --- | --- | --- | --- | --- | --- | --- | --- | --- | --- | --- | --- | --- | --- | --- | --- | --- | --- | --- | --- | --- | --- | --- | --- | --- | --- | --- | --- | --- | --- | --- | --- | --- | --- | --- | --- | --- | --- | --- | --- | --- | --- | --- | --- | --- | --- | --- | --- | --- | --- | --- | --- | --- | --- | --- | --- | --- | --- | --- | --- | --- | --- | --- | --- | --- | --- | --- | --- | --- | --- | --- | --- | --- | --- | --- | --- | --- | --- | --- | --- | --- | --- | --- | --- | --- | --- | --- | --- | --- | --- | --- | --- | --- | --- | --- | --- | --- | --- | --- | --- | --- | --- | --- | --- | --- | --- | --- | --- | --- | --- | --- | --- | --- | --- | --- | --- | --- | --- | --- | --- | --- | --- | --- | --- | --- | --- | --- | --- | --- | --- | --- | --- | --- | --- | --- | --- | --- | --- | --- | --- | --- | --- |
